# Self-replicating digital data storage with synthetic chromosomes

**DOI:** 10.1093/nsr/nwab086

**Published:** 2021-05-14

**Authors:** Xinyu Lu, Tom Ellis

**Affiliations:** Imperial College Centre for Synthetic Biology, Imperial College London, UK; Department of Bioengineering, Imperial College London, UK; Imperial College Centre for Synthetic Biology, Imperial College London, UK; Department of Bioengineering, Imperial College London, UK; Wellcome Trust Sanger Institute, UK

With the rapid development of the Internet and computational technologies, the exponential growth of digital data is beginning to overwhelm existing storage device capacities, driving an unprecedented demand for new data storage media. DNA, a natural genetic information storage system, has been proposed as a promising alternative [1]. Compared with traditional silicon-based storage, DNA-based storage has many advantages—incredible density, long-term stability, low cost of energy to maintain and future-proof compatibility due to our expected need to sequence DNA as long as life exists on Earth. In the past decade, rapid progress in DNA synthesis and sequencing has enabled the ‘writing’ and ‘reading’ of DNA information to drop substantially in cost whilst accelerating in speed [2]. Because of this, synthetic DNA has become an attractive new media to develop, for storing and retrieving data.

Great strides have been made recently in *in vitro* DNA storage, where data are encoded into millions of short synthetic DNA strands stored in tubes, and then read later via DNA sequencing [3]. In comparison, less focus has been on *in vivo* storage systems where the data are stored in DNA in living cells. While this is clearly more complex to achieve it offers an attractive opportunity: cells can replicate thousands of copies of their DNA per day precisely, and at very low cost. So far, the total length of DNA carrying encoded data within a single cell has been limited to just a few thousand base pairs per genome [4]. To break through this limitation, a new study now demonstrates the successful construction of a 254 000 bp artificial chromosome fully dedicated to digital data storage [5].

In this work, Chen *et al.* reported the de novo design and synthesis of a yeast artificial chromosome (YAC) encoding two pictures and a video clip. To convert the digital information into a DNA sequence that is stable *in vivo* and efficient to read by genome sequencing, they employed a clever error correction encoding scheme using sparsed low-density parity-check (LDPC) codes and pseudorandom sequences. The digital files were divided and encoded into bit blocks, which were further successively transcoded into individual synthetic DNA chunks that were physically assembled in yeast into a YAC vector. Interestingly, despite being artificial and non-coding this chromosome was observed to be actively transcribed. Further in-depth research on the transcriptional and translational profiles of this YAC and their effects on the host would advance our understanding of chromosome functions.

As the stability and fidelity of the storage media is a key consideration, the data-carrying YAC was systematically examined through a series of batch cultures and found to be stable in selective growth media; maintained for 100 generations, with no single mutations in the tested samples. Compared with prior work, the overall logical density of encoding in the YAC is 1.19 bits/bp. These results show the viability of using YACs for reliable data storage, a method that can be easily copied.

Whole-genome short read sequencing was efficient at retrieving the data, but the authors also assessed nanopore sequencing, which has the advantages of real-time readout and portability. The high raw error rate (∼10%) of this is a challenge for data recovery, so to solve this problem, a series of correction steps were introduced to mitigate the error rates, with these steps enabled by the LDPC encoding. Their optimized process led to a workflow in which it only takes 11 minutes to sequence the genomic DNA and fully recover the digital data.

**Figure 1.**
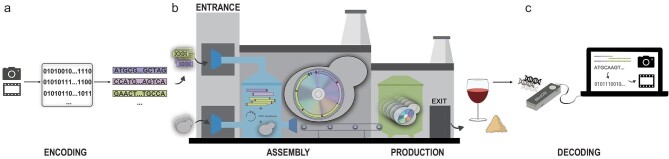
Schematic of yeast chromosome digital data storage concept. (a) Digital files such as pictures and videos are encoded into multiple bit codewords and transcoded into corresponding DNA sequences. (b) Manufacturing of the information storage DNA ‘CD’ starts with the synthesis and assembly of the DNA fragments into a yeast artificial chromosome vector *in vivo*, generating the CD-like data-carrying YAC. The manufactured DNA CD-containing yeast cells can then self-replicate millions of copies and can be easily distributed by incorporation into yeast products. (c) Digital files stored in the YAC can be rapidly retrieved from the long reads generated by a portable nanopore sequencing device.

The work of Chen *et al*. is a key proof-of-concept that demonstrates how artificial chromosomes can be used for data storage in a way that is robust and essentially free to copy. In many ways it is like compact disc (CD) storage where making the original master copy at the factory requires investment, but once made the data can be very easily replicated and shared with others (Fig. 1). It also provides the potential prospect of playing a DNA ‘CD’ any time and anywhere by aligning the work with the capabilities of pocket-sized nanopore sequencing devices. Perhaps it will not be long before an innovative musician shares their latest work by sending samples of yeast to fans with smartphones capable of at-home nanopore sequencing.

***Conflict of interest statement.*** None declared.
